# Supplementary material for: Receptogenesis in a Vascularized Robotic Embodiment
Source: Adv Sci (Weinh). 2026 Aug 7;13(59):e76999. doi: 10.1002/advs.76999 (PMC13449548; doi:10.1002/advs.76999)
Supplement: Supplementary file 1 [file ADVS-13-e76999-s001.pdf]

# Supporting Information for *Receptogenesis in a Vascularized Robotic Embodiment*

*Kadri-Ann Pankratov*, Leonid Zinatullin, Hans Priks, Adele Metsniit, Urmas Johanson, Tarmo Tamm, Alvo Aabloo, Edoardo Sinibaldi*, Indrek Must**

K. A. P., L. Z., H. P., A. M., U. J., Prof. T. T., Prof. A. A., Assoc.Prof. I. M.
Institute of Technology, University of Tartu, Nooruse 1, 50411 Tartu, Estonia

E. S.
Italian Institute of Technology, Via Morego, 30, 16163 Genova, Italy

Email Address: kadri-ann.pankratov@ut.ee, edoardo.sinibaldi@iit.it, indrek.must@ut.ee

This file includes:

- Supporting Information Figures S1 to S4 with captions
- Supporting Movie 1 to 5 descriptions
- Supporting Files 1 to 2 descriptions

# 1 Supplementary Figure S1 - Infill Mediated Distributed Hydraulic System

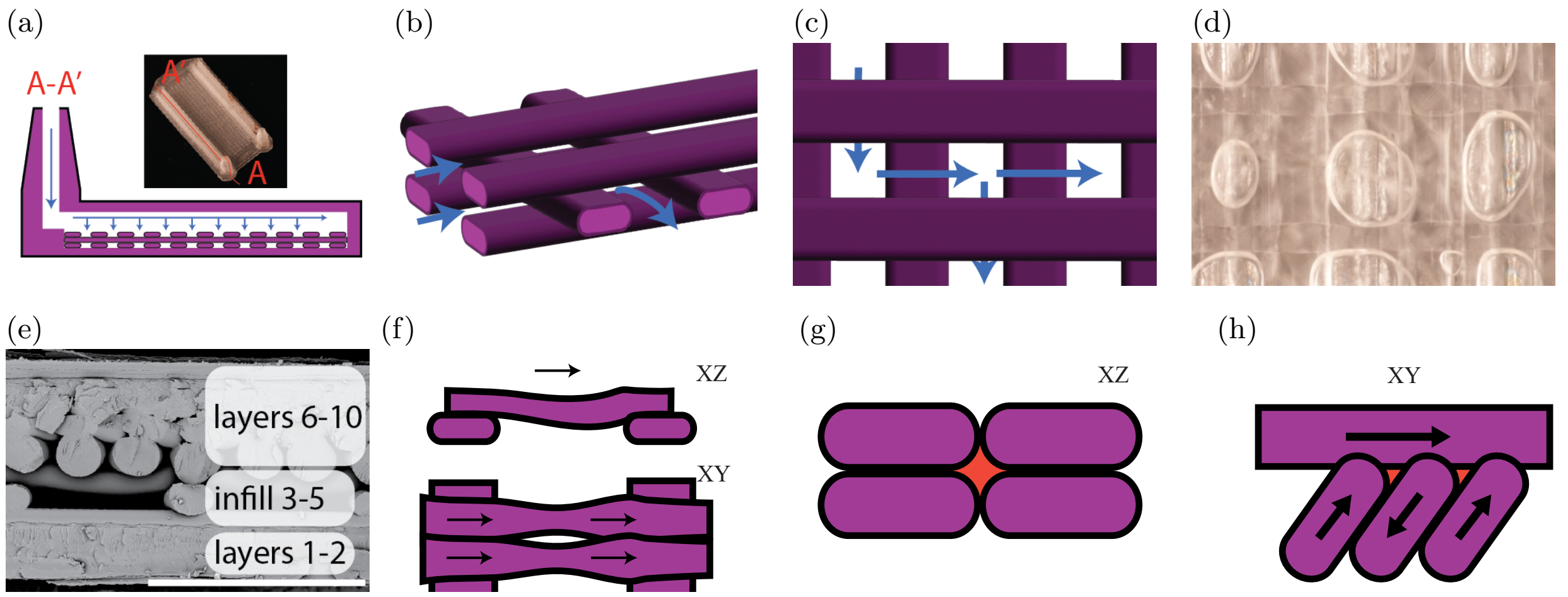


Figure S1: Vascularisation via Fused Deposition Modeling (a) Cross-section of the vascular structure. The liquid enters through the conical hose attachment point, travels down the logistic highway (vein) and enters the distributed permeability structure. (b) Distributed permeability structure consists of print lines placed atop oneanother in a rectilinear pattern. (c) Rectilinear infill is used as system of micro-channels and chambers. Liquid traverses this structure in a taxicab motion. (d) Distributed permeability structure as seen from below. Bubbles were injected to highlight the chambers. (e) Micrograph of the vascular scaffold. Layers 1-2 and 6-10 were printed solid and ironed. Top and bottom layers are leakage proof (f) Bridging between substrates causes an intermittent capillary structure. The sagging of layer 6 produced a capillary-like structure with high relative surface area. (g) Adjacent printing lines produce a star shaped channel (seen in (e) layers 7-8) (h) A common cause of leakage were meeting points of multiple filament lines. This defect was mitigated via ironing. Scale bars: 1 mm.

# 2 Supplementary Figure S2 - Embedded receptor: implementation and demonstration

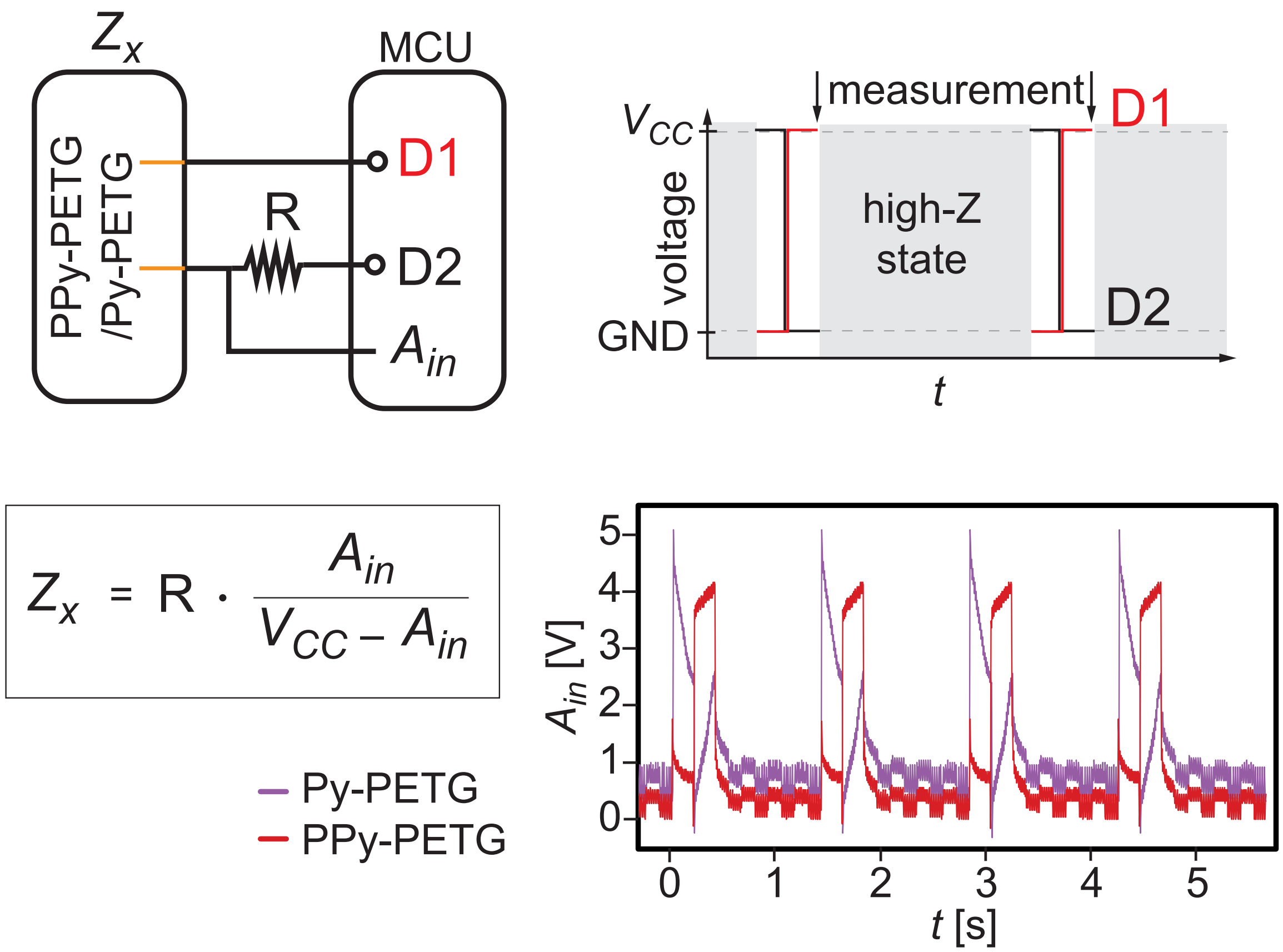


Figure S2: Embedded receptor: implementation using impedimetric readout (left) and demonstration (right). The impedance change was registered by an embedded microprocessor unit featuring a custom bipolar pulse technique combined with voltage division by a constant external resistor. The lower transient voltage across the receptive area (PPy-PETG), versus Py-PETG, showed higher conductance, whereas the lower voltage rate captured an increased capacitance (right, Supplementary Figure S3)

# 3 Supplementary Figure S3 - Low Intensity UV on Py-PETG and PPy-PETG

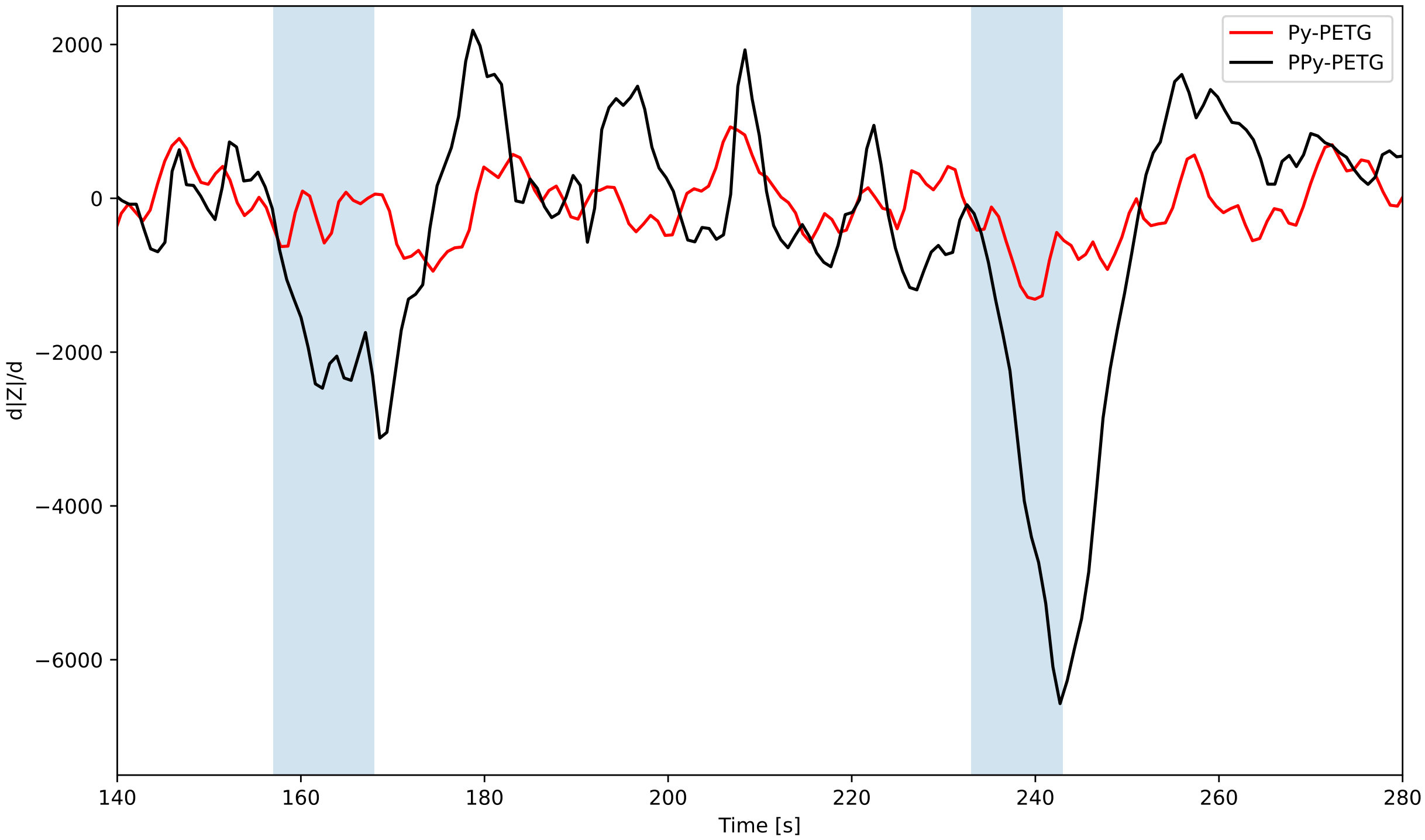


Figure S3: Simultaneous irradiation of Py-PETG and PPy-PETG with a low intensity (0.9 W) UV source (blue area) from a distance of 20 mm shows PPy-PETG reacting to the UV with a reversible decrease in impedance and Py-PETG not showing any significant change in impedance.

## 4 Supplementary Figure S4 - Gold placement

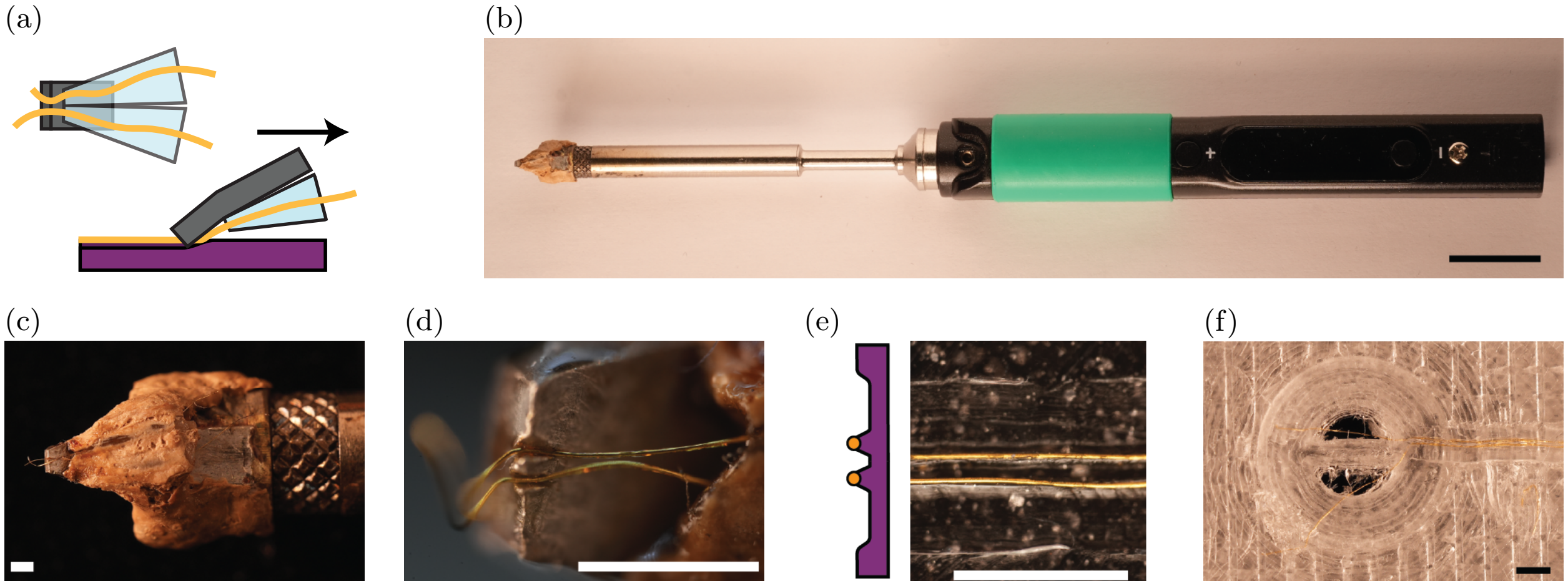


Figure S4: Embedding Gold Electrodes (a) Embedding procedure (b) Embedding tool attached to a soldering iron (c) Embedding tool consisting of glass capillaries bonded to a blade. (d) Silver blade has indentations for guiding the wires (e) Scraper over-moulds the wires with the molten substrate polymer (f) The gold electrodes can be accessed via specialized passthrough. Scale bars: (b) - 10 mm, others 1 mm

## 5 Supplementary Movie 1 - FDM Printed Vascular System

This Supplementary Movie visualizes the FDM printed vascular system fluid flow using air bubbles. As seen from the Movie, liquid is transported throughout the embodiment, with transient bubble patterns inspiring flow-directed programming as in Conway's Game of Life [S1].

## 6 Supplementary Movie 2 - Receptogenesis

This Supplementary Movie shows the transilluminated view of the receptogenesis process. The thorax embodiment is irradiated with UV light while the precursor mixture is introduced via pump-driven advection (pump not shown). Following photopolymerization, the functionality of the receptor is demonstrated by exposing the PPy-PETG composite to UV light, which elicits a visual signalling response indicated by flashing LEDs.

## 7 Supplementary Movie 3 - Circulatory Fluids in the Artificial Embodiment

This Supplementary Movie demonstrates the artificial robotic circulatory system. An empty embodiment is first filled with isopropanol, which is subsequently exchanged with a blue-traced isopropanol:water mixture to visualize fluid flow.

## 8 Supplementary Movie 4 - Robotic Demonstration of Receptogenesis

This Supplementary Movie demonstrates receptogenesis in a moth-inspired robotic platform. First, the receptive area is generated within the thorax, triggering a wing-flap response from the embodiment. Second, the receptive area is generated within the wing, during which pump-driven advective flow of the precursor mixture is visible.

## 9 Supplementary Movie 5 - *Ex novo* Receptor Demonstration

This Supplementary Movie demonstrates the activation of the receptors. First, activation of the wing receptor is shown alongside its corresponding visual signal (flashing red LED). Second, activation of the thorax receptor and the corresponding wing-flap response are demonstrated. Subsequently, both wing and thorax receptors are activated, followed by the introduction of circulatory fluid, after which the thorax receptor is successfully re-activated. A

control receptor lacking a receptive area (plain PETG) is then irradiated with UV light, confirming the absence of activation. Finally, thorax activation and the consecutive wing-flap response are shown from a side view.

## 10 Supplementary file 1. Printing configuration Bambu A1

This supplementary file contains the OrcaSlicer project file including all printed fluidic structures: two wings, one test wing, the thorax, and the body. Each component is configured with representative print settings optimized for the Bambu A1 printer. Gold embedding requires the insertion of an additional pause command at layer 3.

## 11 Supplementary file 2. Printing configuration Prusa XL

This supplementary file contains the OrcaSlicer project file including all printed fluidic structures: two wings, one test wing, the thorax, and the body. Each component is configured with representative print settings optimized for the Prusa XL printer. Gold embedding requires the insertion of an additional pause command at layer 3.

## References

[S1] Martin Gardner. MATHEMATICAL GAMES. *Scientific American*, 223(4):120–123, 1970. ISSN: 00368733, 19467087. URL: http://www.jstor.org/stable/24927642.
